# Chemical modification of bacterial exopolysaccharides: Antioxidant properties and health potentials

**DOI:** 10.1016/j.crfs.2024.100824

**Published:** 2024-08-18

**Authors:** Irshad Ahmad Shah, Digambar Kavitake, Swati Tiwari, Palanisamy Bruntha Devi, G. Bhanuprakash Reddy, Krishna Kumar Jaiswal, Amit K. Jaiswal, Prathapkumar Halady Shetty

**Affiliations:** a Department of Food Science and Technology, Pondicherry University, Pondicherry, 605014, India; b Biochemistry Division, ICMR – National Institute of Nutrition, Hyderabad, 500007, India; c Bioprocess Engineering Laboratory, Department of Green Energy Technology, Pondicherry University, Puducherry, 605014, India; d School of Food Science and Environmental Health, Faculty of Sciences and Health, Technological University Dublin – City Campus, Central Quad, Grangegorman, Dublin D07 ADY7, Ireland

**Keywords:** *Bacteria*, *Exopolysaccharides (EPS)*, *Chemical modifications*, *Antioxidant potential*, *Health benefits*

## Abstract

In recent years, there has been a burgeoning interest in the utilization of microbial exopolysaccharides (EPS) because of the added advantage of their renewable, biocompatible, and biodegradable nature in addition to intended applications. The endowed properties of bacterial EPS make them valuable candidates for a wide array of industrial applications. Modification of native EPS is known to enhance various physico-chemical and functional properties. Various modifications such as physical, chemical, biological, and enzymatic modifications were practiced improving the bioactivity of EPS. This paper comprehensively aims to review the most recent chemical modification techniques employed to modify the physico-chemical and functional changes of bacterial EPS in comparison with the unmodified forms. Chemical modification entails strategic alterations to the structure and properties of EPS through various synthetic and semi-synthetic methodologies. Emphasis is given to the antioxidant potential and functional role of these EPS derivatives in human health. Antioxidant properties reveal a significant augmentation in activity compared to their native counterparts. Such enhancement holds a strong promise for potential benefits and therapeutic applications. Chemical derivatives of EPS with overwhelming functional benefits could surely encourage EPS application, particularly as potential hydrocolloids in industrial and biomedical contexts.

## Highlights

• The article reviews chemical modifications, like sulfation and acetylation, to enhance EPS properties.
• Modified EPS have higher antioxidant properties, boosting scavenging and therapeutic benefits.
• Highlights health benefits of modified EPS as antimicrobial, antiviral, anticancer, and prebiotic.
• EPS modifications promise to treat oxidative stress, cancer, cardiovascular issues, and inflammation.
• Enhanced antioxidant properties make modified EPS valuable for various therapeutic applications.

## Graphical abstract

**Figure:**
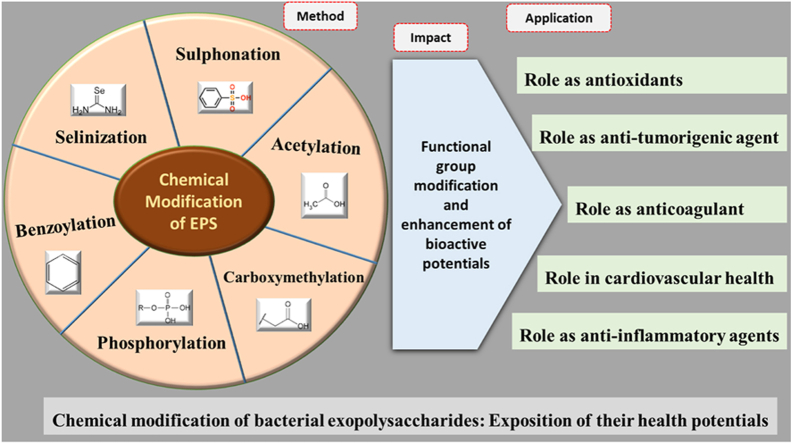

## 1 Introduction

Exopolysaccharides (EPS) are polysaccharides secreted into the environment by various microorganisms. These polymers can be obtained from microbes like archaea, bacteria, and fungi (Chaisuwan et al., 2020). Bacteria synthesize EPS as a means to survive under unfavorable conditions of nutrient stress, temperature fluctuations, and toxic compounds (Mathivanan et al., 2021). Synthesis of EPS occurs through an intracellular sequence of events via enzymes and is later exported to its surrounding environment (Cui et al., 2017). EPS are biological macromolecules consisting of repeating units of sugars that hold unique physicochemical attributes and rheological properties with a GRAS (generally regarded as safe) status (Yildiz and Karatas 2018; Kavitake et al., 2024). Bacterial-origin polysaccharides offer several advantages over polysaccharides derived from plants or animals, including higher yields in smaller time intervals, superior product quality, and controlled, renewable production processes that minimize environmental impact (Moscovici 2015). They play a role as antimicrobial, antiviral, anticancer, prebiotic, and cholesterol-lowering agents (Yildiz and Karatas 2018; Devi et al., 2021; Kavitake et al., 2023). Besides that, they have numerous applications in food, agriculture, biomedical, and pharmaceutical sectors (Riaz et al., 2021).

EPS modification has captured special attention very recently from the scientific community. Polymer modification is a targeted tool to add novel additives to food systems, through physical, chemical, biological, and enzymatic means. Chemical derivatives of EPS can be formed through sulfation, acetylation, carboxymethylation, phosphorylation, benzoylation (Liu et al., 2012), and selenization (Li et al., 2020) procedures. Polymers through acetylation possess greater water solubility, swelling capacity, and clearer paste upon gelation (Ayucitra 2012). Acetylated polymer produced from *Ganoderma atrum* exhibited stronger radical scavenging ability, caused inhibition in Beta-carotene linoleic systems, and elevated potential for macrophage phagocytosis and tumor necrosis factor secretions (Chen et al., 2014). Applications of these modified polymers have been expanded in baked, canned, and frozen foods like fruit pies, gravies, filled cakes, and salad dressing (Ayucitra 2012). They perform other desirable applications in the field of metal complexation, organic catalysis, and chiral separation (Cho et al., 2014).

## 2 Bacterial EPS structure

EPS produced by bacteria have a diverse chemical structure that plays a significant role in various physicochemical and biological properties. Their applicability relies on their chemical structure, making it essential to determine the monosaccharide compositions, molecular weights, linkage types, and fine structures of these EPS. Scientists have categorized EPS into homopolysaccharides (HoPs) and heteropolysaccharides (HePs). HoPs are composed of single types of monosaccharides like glucose and fructose that are connected through glycosidic bonds. HePs, on the other hand, contain two or more units of different monosaccharides like glucose, galactose, fructose, fucose, rhamnose, mannose, N-acetylglucosamine, and uronic acids (Mohd Nadzir et al., 2021). Polymers included in the category of heteropolysaccharides include xanthan gum, alginate, hyaluronic acid, kefiran, and gellan (Netrusov et al., 2023). The majority of EPS produced belong to heteropolysaccharides (HePs), synthesized intracellularly, while homopolysaccharides are produced extracellularly using the enzymatic machinery (Lynch et al., 2018). HePS are synthesized in limited quantities by lactic acid bacteria. However, these demonstrate significant thickening capabilities even at low levels of concentration (Patel et al., 2012). For instance, Kefiran, a heteropolysaccharide produced by *Lactobacillus kefirgranum*, *Lactobacillus kefiranofaciens* is branched glucogalactan. It has a complex structure composed of D-glucose and D-galactose in almost equal proportions. The pattern of linkage is glucose linked by (1 → 6) bonds, galactose linked by (1 → 3) bonds, galactose linked by (1 → 4) bonds, glucose linked by (1 → 4) bonds, and galactose linked by (1 → 2, 6) bonds (Moradi and Kalanpour 2019). Their reports infer that these specific linkages make such compounds resistant to digestive enzyme hydrolysis, but they can be beneficial for the colon while undergoing fermentation. In general, bacterial α-glucans exhibit a great deal of structural variety. They are made up of glucopyranoses that connect to one other along a backbone via 1,3, 1,4, or 1,6 linkages (Munkel et al., 2019). Water solubility is greatly affected by the backbone structure. Glucans become insoluble in water when they have a specific amount and distribution of α-1,3-linkages, although dextrans (glucan having 1,6 linked backbone) retain their solubility. It was demonstrated that 1,3-linkages appear to be a critical component for water insolubility, while the precise criteria are still obscure (Wiater et al., 2012b).

The molecular weight of EPS significantly influences their physical, chemical, and functional properties. Understanding these effects is essential for tailoring EPS for specific applications in sectors like food, pharmaceutical, and biotechnology. EPS secreted by *Lactobacillus helveticus* is composed of high molecular weight (5.21 × 10^6^ Da), medium molecular weight (2.39 × 10^6^ Da) and low molecular weight (3.76 × 10^5^ Da). The higher molecular weight greatly enhances the interaction with proteins, leading to a tighter gel network by filling the spaces within casein clusters in fermented milk (Zhang et al., 2024). Thus, high molecular-weight EPS in yogurt manufacturing can enhance product quality and drive industrial growth. Akkerman et al. reported that fructan EPSs comprised of low, medium, and high molecular weight exhibit immunomodulatory activity in dose-dependent patterns. However, TLR2 signaling gets more strongly stimulated with high molecular weight levans (Akkerman et al., 2024). This paves the way for using β-(2,6)-fructans as an immunomodulating component in conditions involving TLR signaling, such as mucositis, allergies, and other intestinal disorders.

## 3 Modification of EPS

Molecular modification is considered an important area of polysaccharide chemistry. These chemical modifications have a role in enhancing or augmenting the bioactivities of natural polysaccharides that have lower biological activity. Therefore, it turns out to be very attractive to investigate the effect of alterations in the bioactivity of microbial EPS via molecular modifications. Methods of modification broadly comprise physical, biological, and chemical modifications. These modifications alter the biological activity of polysaccharides by changing their structure, molecular weight, and functional groups. Biological and physical alterations only modify the molecular weight of polysaccharides, whereas chemical alterations can modify polysaccharide substituents (Nachtigall et al., 2021). EPS from various bacterial sources and their influenced properties after chemical modification are depicted in (Table 1). Therefore, a summary of the relationship between bioactive potential and their functional role due to such modifications need to be reported. This could enable the researchers and food product developers to harness applications for specific food use.

**Table 1:** Exopolysaccharides from bacterial sources with types of chemical modification and their influenced properties.

EPS/sugar components	Bacterial strain	Source/origin of isolation	Type of modification	Key properties influenced	References
Levan	*Bacillus subtilis* NRC1aza	Mediterranean Sea shallow water	Sulfation	Strong free radical scavenging activity with DPPH, antitumor activity of SL1 against different human cancer cell lines, Cytotoxicity against human cell lines hepatocellular carcinoma HepG2 cells, induced intrinsic apoptosis pathway in liver cancer cells.	Abdel-Fattah et al. (2012)
Levan	*Paenibacillus polymyxa* EJS-3	Root tissue of *Stemona japonica* (Blume) Miquel, traditional Chinese medicine	Acetylated, phosphorylated and benzoylated	Higher anti-proliferative activity against human gastric cancer BGC-823 cells, promising antioxidant and antitumor agents than native EPS.	Liu et al. (2012)
Glucose, galactose, galacturonic, glucuronic	*Alteromonas infernus*,	Originated from a deep-sea hydrothermal vent	Sulfation	Anticoagulant activity observed in sulfated fraction. Inhibited osteoclast genesis in bone marrow stem cell models, reduced proliferation and accelerated osteoplastic differentiation	Guezennec et al. (1998) Ruiz Velasco et al. (2011)
(1 → 6)-α-dextran	*Leuconostoc* spp	Not mentioned	Carboxymethylation	Stronger scavenging capacity against OH radicals but lower scavenging capacity for DPPH radicals compared to that of pure dextran, highest glass transition temperature	Li et al. (2021)
Galactose, glucose and mannose	*Lactobacillus helveticus* MB2-1	Ropy fermented milk (Sayram) from Xinjiang of China	Acetylated, carboxymethylated and sulfated	Thermal properties and surface morphology were greatly changed. Antioxidant activities were enhanced after these modifications. However, sulphation contributed greatly toward chelation of ferrous ions and scavenging activity of radicals.	Xiao et al. (2020)
Glucose, galactose, and fucose and Glucuronic acid	*Enterobacter* sp. ACD2	Marine environment of Haqel Beach in the Tabuk region of Saudi Arabia	Sulfation	Sulfated EPS exhibited higher antibacterial activity against *Staphyllococcus aureus* and *Escherichia coli*. fibrinolytic and anticoagulation properties were also observed.	Almutairi and Helal (2021)
Glucose, mannose and galactose	*Lactobacillus plantarum 70810*	Chinese Paocai	Acetylation, carboxymethylation phosphorylation and	Compared with native EPS, the chemical derivatives exhibited larger antioxidant and antitumor activities.	Wang et al. (2015)
Tetrasaccharide of repeating unit composed of two aminosugars (N-acetyl-glucosamine, GlcNAc and N-acetyl-galactosamine, GalNAc) and two glucuronic acid (GlcA) units	*Vibrio diabolicus* HE800	Marine Deep-sea hydrothermal vents, originally isolated from the polychaete annelid Alvinella pompejana in the East Pacific Rise	Sulfated	Bone regeneration capacity, revealing to act as an efficient filler of bone defects in rat calvaria, without showing any inflammatory activity. persulfated derivative of HE800 EPS exhibit biological activities similar to sulfated glucosamine glycan's (GAGs) such as heparin and heparan sulphate	Esposito et al. (2022)
Se-EPS	*Lactococcus lactis* subsp. *lactis*	Fermented food	Selenization	Se-EPS enhanced phagocytosis and increased nitric oxide (NO), IL-12, IL-6, IL-1, except IL-10 levels in mouse peritoneal macrophages. Invitro tests in mouse spleen revealed that Se-EPS stimulated mouse spleen lymphocyte proliferation, and markedly raised mRNA levels of TNF-α, IL-2 and IL-6 in spleen cells. Compared to native EPS, Se-EPS exhibited stronger immunomodulatory action.	Pan et al. (2015)
Se-EPS	*Lactococcus lactis* subsp. *lactis*	Fermented foods	Selenization	In lymphocytes, Se-EPS enhanced the levels of expression and phosphorylation of Ca2+-calmodulin-dependent kinase II. Increased lymphocyte proliferation and activation was witnessed through calcium signaling due to Se-EPS. Se-EPS at lower dose has potential to activate PKA, the calcium channel, NO, and cAMP pathways.	Liu et al. (2013)

### 3.1 Physical modification

The application of external energy to break down glycosidic connections in order to lower polysaccharide molecular weight is known as physical modification (Chaisuwan et al., 2020). Ultrasonic disruption, thermal, alkaline, radiation-induced reaction, and microwave exposure are commonly used techniques (Viñarta et al., 2013; Li et al., 2016). Ultra sonication of 20-kHz at 60 % amplitude causes a reduction in molecular weight in konjac glucomannan, which is associated with random chain scission and midpoint chain scission mechanism (Yin et al., 2019). Size reduction was evidenced through the decrease in the hydrodynamic and zeta-average radius. Thermal and alkaline treatment in scleroglucan caused a decline in apparent viscosity and loss of pseudo-plastic nature (Viñarta et al., 2013). Suitable ultra-sonication parameters (amplitude and duration) proved to be a likely approach for adjusting the viscosity and molecular mass of EPS (Nachtigall et al., 2021). EPS of *W. confusa* TISTR 1498 showed better immune-enhancing potential when modified with HCl and microwave/boiling (Chaisuwan et al., 2020).

### 3.2 Biological modification

In biological modification, enzymes accelerate the breakdown of polysaccharides to their molecular weight forms. These biological alterations have a high level of selectivity and efficiency (Whitfield et al., 2015). However, the enzyme can only catalyze particular kinds of polysaccharides which appeared as a limitation (Chaisuwan et al., 2020). Functional groups like succinyls, acetyls, glyceryls, pyruvyls, and dactyls from EPS can be introduced or eliminated by transferases and hydrolases that can affect the surface electrostatics and solubility of the polymer (Whitfield et al., 2015).

### 3.3 Chemical modification

It is a typical approach of modification that involves inserting substituent groups into the structure of polysaccharides, therefore boosting their bioactivities (Zhang et al., 2016). Despite the structure of a polysaccharide is tied to its functions and qualities, chemical changes can modify the microstructures of polysaccharides to boost biological activities. Chemical changes include sulfation, acetylation, carboxymethylation, phosphorylation, benzoylation and selenization (Chaisuwan et al., 2020).

### 3.4 Types of chemical modifications

#### 3.4.1 Sulfation

**Fig. 1:**
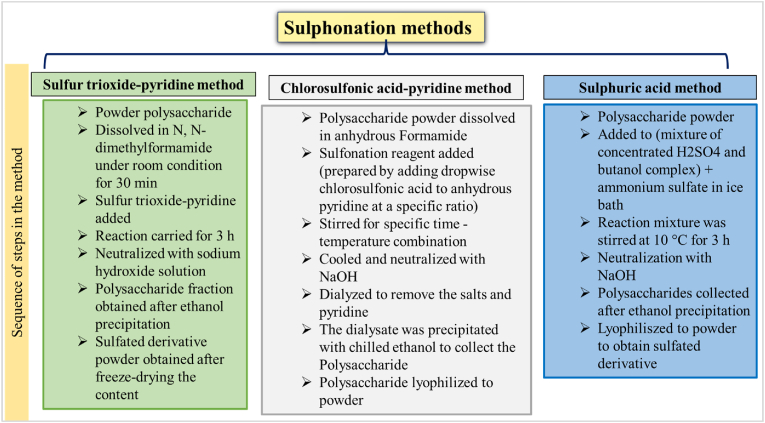
Sulphonation methods and sequence of steps during the process.

The substitution of sulphate groups for the hydroxyl, carboxyl, and amino terminals of polysaccharides resulted in sulfated exopolysaccharides (Andrew and Jayaraman, 2020). Commonly used methods for the synthesis of sulphonated derivatives are sulfur trioxide-pyridine method using (*N*, *N*-dimethylformamide (DMF), SO_3_·Py) (Sun et al., 2015), chlorosulfonic acid-pyridine method (Chlorosulfonic acid (CAS), anhydrous pyridine) (Xu et al., 2016) and sulfuric acid method (Sulfuric acid, butanol complex, ammonium sulphate) (Jiang et al., 2014) and the sequence of steps involved in the synthesis process are presented in (Fig. 1).

Each method has its intent for the use like, for synthesis of higher substituted derivatives sulfur trioxide-pyridine performs better. The degree of sulphation is determined by barium chloride turbidimetry (Liu et al., 2021). These highly substituted polymers with a higher degree of substitution are correlated with enhanced bioactivities (Lu et al., 2008). The sulfuric acid method has the advantage of establishing a stable reaction environment with low toxicity of chemicals over the chlorosulphonic acid. However, carbonization and degradation of polymers can happen which is considered a drawback in the sulfuric acid method (Jiang et al., 2014). It was reported that EPS from *Streptococcus thermophilus* when subjected to the sulphonation process, a minimal change in microstructure, a decline in degradation temperature, and enhanced solubility observed. Furthermore, antioxidant, antiviral, antitumor, anticoagulation, and antibacterial properties were noted (Zhang et al., 2016). Sulfated EPS are in the limelight for their enhanced bioactivities. The maximum antioxidant potential of various sulfated EPS from bacterial sources is depicted in Table 2 showing the concentration of EPS and percentage inhibitions for different antioxidant assays. To mention one example, EPS from *Streptococcus thermophilus* GST-6 showed concentration-dependent DPPH inhibition which was significantly (P ≤ 0.05) higher (18.29 %) for sulfated than unmodified (9.26%) at (1.0 mg/mL) (Zhang et al., 2016). However, at the same concentration of 1.0 mg/mL sulfated EPS from *Streptococcus thermophilus* ASCC 1275 presented almost two fold percent inhibition (14.55 %) than unmodified (7.71%) (Li and Shah 2014). More recently, fungal EPS have also been explored for biological activities although it is beyond the scope of the review. To discuss a few of them, for example, synthesized sulfated glucan EPS isolated from *Penicillium* sp*.*gxwz446 in south China exhibited significant dose-dependent pinocytic activity of RAW264.7 cells and triggered NO production. It was observed that sulfated EPS also presented a higher phagocytosis rate of 65.1 % than unmodified 50.5 % at 0.2 mg/mL (Sun et al., 2016). More recently (Wu et al., 2023) reported naturally occurring marine fungus *Aspergillus versicolor* SCAU141 synthesized sulfated EPS showed enhanced activity of TNF-α, COX-2 and better immune activity in RAW264.7 macrophage by activating the (NF- κB/p65 and MAPK/p38) specific pathway. Immunomodulatory activity they proved by metabolomics was closely resembling to arginine synthesis amino acid metabolism (Wu et al., 2023). These EPS are reported to hold the capacity to donate hydrogen due to low dissociation energy of O-H bond (Jin et al., 2011).

**Table 2:** Antioxidant activity of chemically modified EPS derivatives.

EPS producing Strain	Tested dose of EPS	Antioxidant capacity of various chemically modified derivatives of EPS					Degree of substitution (DS)	References
		DPPH (%)	Superoxide radical scavenging (%)	Hydroxyl radical scavenging (%)	FRAP	Metal/chelation/Lipid peroxide inhibition (%)	DS	References
**Antioxidant activity of sulfated EPS**								
*Streptococcus thermophilus* GST-6	1.0 mg/mL	18.29 for Sf; 9.26 for Um; 34.72 for AA	48.24 for Sf; 34.63 for Um; 45.01 for AA	59.22 for Sf; 36.47 for Um; 51.37 for AA	–	–	0.26	Zhang et al. (2016)
*Streptococcus thermophilus* ASCC 1275	1.0 mg/mL	10.07 for Sf; 7.40 for Um;	19.66 for Sf; 12.65 for Um;	12.63 for Sf; 7.85 for Um	–	–	0.31	Li and Shah (2014)
*Pleurotus eryngii*	1.0 mg/mL	14.55 for Sf; 7.71 for Um;	35.10 for Sf; 22.33 for Um;	23.44 for Sf; 21.81 for Um	–	–	0.69	Li and Shah (2014)
*Lactobacillus helveticus MB2-1*	4.0 mg/mL	59.5 for Sf; 38.0 for Um; 89.0 for vit C	60.5 for Sf; 50.0 for Um; 75 for vit. c	70.0 for Sf; 99 for Um; 55.5 for vit c	–	Metal ion chelation 72 for Sf; 45.5 for Um; 99 for vit c	0.625	Xiao et al. (2020)
*Lactobacillus plantarum BR2*	2.0 mg/mL	Approx. 64.75 for Sf; 61.0 for Um	–	Approx. 55.0 for Sf; 35.0 for Um; 70.0 for vit c	41.0 for Sf; 15.0 for Um	–	–	Soumya and Nampoothiri (2023)
*Lactobacillus plantarum* ZDY2013	2.0 mg/mL	20 for Sf; 2.5 for Um; 100 for Vit. c	Approx. 55 for Sf; 48 for Um; 90 for vit.c	Approx. 67 for Sf; 55 for Um; 90 for vit.c	–	–	0.29	Zhang et al. (2016)
*Enterobacter cloacae* Z0206	2.0 mg/ml	–	Approx.85.06 for Sf; 30.0 for Um 80.66% for vit.c	Approx. 29.2 % for Sf; 12 for Um; 98 for vit.c	–	–	0.60	(Jin et al., 2011)
*Enterobacter cloacae* Z0206	2.0 mg/ml	–	Approx. 89.80 for Sf; 30.0 for Um; 80.66% for vit.c	Approx. 31.5 for Sf, 12 for Um; 98 for vit.c	–	–	0.68	(Jin et al., 2011)
**Antioxidant activity of acetylated EPS**								
*Lactobacillus helveticus* MB2-1	4 mg/mL	45.0 for Ac; 38.0 for Um; 89.0 for vit C	55.0 for Ac; 50.0 for Um; 75.0 for vit c	59.5 for Ac; 55.5 for Um; 99.0 for vit c	–	Metal ion chelation 61.0 for Ac; 45.5 for Um; 99.0 for vit c	0.439	Xiao et al. (2020)
Lactobacillus plantarum BR2	2.0 mg/mL	73.81 for Ac; 61.0 for Um	–	Approx. 40.0 for Ac; 35.0 for Um; 70.0 for vit c	Reducing power 37.0 for Ac; 15.0 for Um	–	–	Soumya and Nampoothiri (2023)
*Paenibacillus polymyxa EJS-3*	1.0 mg/mL	–	77.8 for Ac; 40.4 for Um; 12.11 for Vit.c	80.26 for Ac; 68.55 for Um	–	–	0.53	Liu et al. (2012)
*Lactobacillus plantarum* 70810	4.0 mg/mL	49.5 for Ac; 28.18 for Um	–	58.48 for Ac; 30.46 for Um	–	–	0.41	(Wang et al. 2015)
**Antioxidant activity of carboxymethylated EPS**								
*Lactobacillus helveticus* MB2-1	4.0 mg/mL	62.0 for Cm; 38.0 for Um; 89.0 for vit C	55.5 for Cm; 50.0 for Um; 75.0 for vit c	79.0 for Cm; 55.5 for Um; 99.0 for vit.c	–	Metal ion chelation 68.0 for Cm; 45.5 for Um; 99.0 for vit c	0.526	Xiao et al. (2020)
Lactobacillus plantarum BR2	2.0 mg/mL	63.78 for Cm; 61.0 for Um	–	Approx. 49.0 for Ac; 35.0 for Um; 70.0 for vit.c	Reducing power 24.0 for Ac; 15.0 for Um	–	–	Soumya and Nampoothiri (2023)
*Lactobacillus plantarum* 70810	4.0 mg/mL	36.5 for Cm; 28.18 for Um	–	78.67 for Cm; 30.46 for Um	–	–	1.1	(Wang et al. 2015)
*Leuconostoc* spp	3.0 mg/mL	27.0 for Cm; 58 for Um	∼10.09 for Cm; 5.09 for Um	17.65 for Cm; 15.20 for Um	–	–	0.57	Li et al. (2021)
*Leuconostoc* spp	3.0 mg/mL	∼22.5 for Cm; 58.0 for Um	∼10.4 for Cm; 5.09 for Um	51.52 for Cm; 15.20 for Um	–	–	0.78	Li et al. (2021)
*Leuconostoc* spp	3.0 mg/mL	20.5 for Cm; 58.0 for Um	∼11.01 for Cm; 5.09 for Um	74.31 for Cm; 15.20 for Um	–	–	1.13	Li et al. (2021)
*Leuconostoc* spp	3.0 mg/mL	18.5 for Cm; 58.0 for Um	∼11.9 for Cm; 5.09 for Um	93.68 for Cm; 15.20 for Um	–	–	1.25	Li et al. (2021)
**Antioxidant activity of phosphorylated EPS**								
*Lactobacillus helveticus* MB2-1	4 mg/mL	65.52 for Pr; 38.0 for Um; 89.0 for vit C	58.0 for Pr; 50.0 for Um; 75.0 for vit c	64.5 for Pr; 55.5 for Um; 99.0 for vit c	–	Metal ion chelation 63.0 for Pr; 45.5 for Um; 99.0 for vit c	0.432	Xiao et al. (2020)
*Lactobacillus plantarum* 70810	4.0 mg/mL	54.95 for Cm; 28.18 for Um	–	31.5 for Cm; 30.46 for Um	–	–	0.08	(Wang et al. 2015)
*Paenibacillus polymyxa EJS-3*	1.0 mg/mL	–	47.3 for Pr; 40.4 for Um; 12.11 for vit. c	89.47 for Pr; 68.55 for Um		–	0.89	Liu et al. (2012)
**Antioxidant activity of benzoylated EPS**								
*Paenibacillus polymyxa* EJS-3	1.0 mg/mL	–	89.8 for Bz; 40.4 for Um	72.37 for Bz; 68,55 for Um	–	–	–	Liu et al. (2012)

**Antioxidant activity of Selenized EPS**								
*Bacillus licheniformis*	4.0 mg/mL	∼39.3 for Sn; ∼39.7 for Um; ∼100 for vit. c	∼72.0 for Sn; ∼55.0 for Um; ‘∼100 for vit. C	∼79.5 for Sn; ∼73.4 Um; ∼100 for vit. C	–	–	–	Wang et al. (2023)

Where, Sf = sulfated EPS; Ac = acetylated EPS; Cm = carboxymethylated EPS; Pr = phosphorylated EPS, Bz = benzoylated EPS; Sn = selenized EPS; Um = unmodified; AA = Ascorbic acid (Positive control); vit.c = Vitamin C (+ve control).

#### 3.4.2 Acetylation

**Fig. 2:**
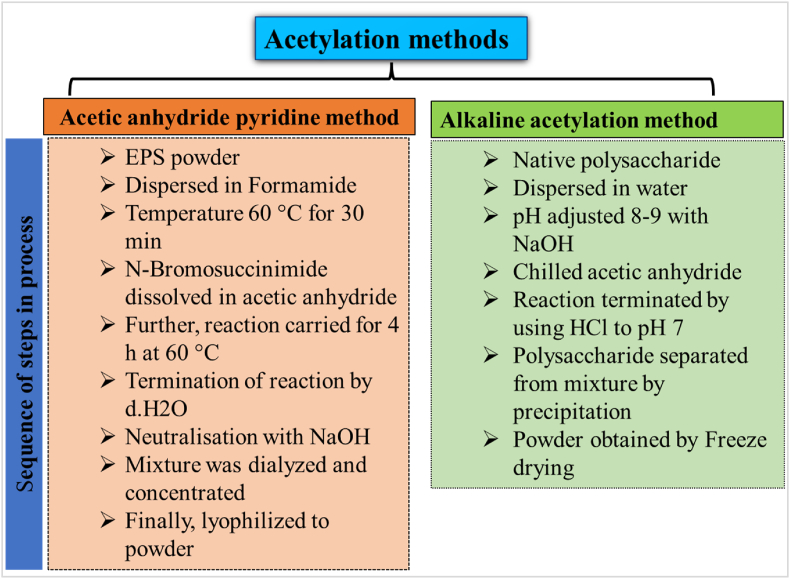
Sequence of steps in the acetylation process for EPS modification.

Acetylation is an inexpensive and environmentally friendly approach to derivitisation (Zhang et al., 2013). The acetylation converts –OH (hydroxyl) groups present on the sugar unit of EPS to acetyl groups (-COCH3) by the esterification reaction. Activation of anomeric carbon and weakening of hydrogen bonds can be ensured through proper acetylation (Wang et al., 2015). This modification has been more prominent in plant polysaccharides and alters cell wall structure and function especially, protecting them from damage caused by hydrolytic enzymes of microbes (Berti et al., 2018). The process of acetylation could bring changes in chain conformation, unmask more polar groups, modify bioavailability and bioactivity, and enhance the water solubility of polysaccharides (Xiao et al., 2019). Acetylation having paramount implications can be incorporated through various methods. A sequence of steps involved in each method depicted in (Fig. 2) are broadly grouped into (a) acetic anhydride pyridine method involving reactions in a non-aqueous medium and (b) alkaline acetylation method in aqueous medium (Biswas et al., 2018). As a result, hydroxyl groups are changed to ethanoates, and various reaction factors like temperature, reaction duration, acetic anhydride concentration, and solvent types impact the degree of substitution (DS) and polysaccharide properties (William N. Sanchez Luna et al., 2018). The mean number of substituted hydroxyl groups per anhydro-glucose unit can provide the degree of substitution value (Ogawa et al., 1999). Based on (DS) acetylated polysaccharides are categorized as low (0.01–0.2) having the properties as stabilizers, thickeners, texturizers, and film-forming agents (Boutboul et al., 2002), intermediate (0.2–1.5), or high (DS) (1.5–3) with a potential of thermoplasticity and solubility in acetone or chloroform (Elomaa et al., 2004; Colussi et al., 2014). Low substituted polysaccharides are obtained after esterification in the aqueous medium where NaOH, alkali, and alkali earth metals act as catalysts (Wang and Wang 2002). The introduction of hydrophilic substituted groups might be a cause of altered swelling and solubility in polymer granules during acetylation (Das et al., 2010). Thermal stability, viscosity, and emulsification properties of acetylated polysaccharide nanomaterial were affected (Lee et al., 1996; Halahlah et al., 2022).

The polysaccharide's antioxidant capacity is attributed to a number of mechanisms, including the breakdown of peroxides and super-oxides, chain initiation (CI), radical capture, single electron transfer (SET), chelating transition state metal ions, and hydrogen atom transfer (HAT) potential (Liu et al., 2012b). Broadly, the antioxidant prospect of polysaccharides relies on structural configuration, extent of substituted groups, branching pattern, monosaccharide composition, molecular weight, and functional groups (Chen et al., 2014; Yuan et al., 2019). There is a scarcity of studies on the modification of lactic acid bacterial exopolysaccharides. However, reports of EPS from *Lactobacillus plantarum* BR2 showed improved biological properties after acetylation (Soumya and Nampoothiri 2023). *Lactobacillus plantarum* BR2 EPS showed DPPH radical scavenging, total antioxidant, and ferric-reducing power in dose dose-dependent manner. Acetylated EPS showed the highest DPPH inhibition at all tested concentrations (0.15–2.0 mg/mL) with a maximum of 73.81 % and unmodified showed 61.0 % at 2.0 mg/mL respectively. Total antioxidant activity (TAA) potential was increased four-fold after acetylation. On the other hand, a 0.7-fold decrease in cholesterol activity was observed compared to control (unmodified) EPS (Soumya and Nampoothiri 2023). One of the unrelated study (Chen et al., 2014) conducted on fungal polysaccharides from *Ganoderma atrum* belonging to the Basidiomycota family showed enhanced activity to scavenge DPPH radical, inhibiting activities in βeta-carotene–linoleic acid systems in comparison to the native form. Furthermore, increased macrophage potential and tumor necrosis factor TNF-α secretions were reported. During inflammation, macrophages largely produce TNF-a, a potent pro-inflammatory cytokine. It plays a vital function in controlling immunological reactivity, either independently or in conjunction with other cytokines, and was revealed to be a mediator of damage during autoimmune and infectious diseases of CNS (central nervous system) (Chen et al., 2014). The acetylation process of polysaccharides could be a fascinating area of research with significant implications in food protection, texture modification, control release of bioactive, and fat replacement in various food and pharmaceutical sectors.

#### 3.4.3 Carboxymethylation

**Fig. 3:**
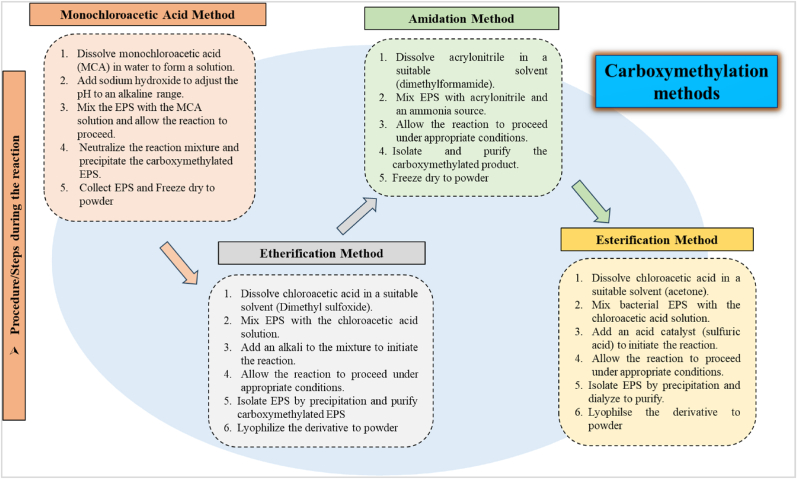
Sequence of steps in carboxymethylation of bacterial EPS.

This modification relies on the Williamson synthesis, during which the polymer combines with the mono-chloroacetic acid so the hydroxyl (-OH) groups are changed to carboxylic groups (Santos et al., 2019). Carboxymethylation of polysaccharides is a topic of extensive research. Since it is a simple chemical modification accomplished through the reaction of the polymer with chloroacetic acid. There are various methods to obtain carboxymethylated EPS derivatives as depicted in (Fig. 3). Briefly, EPS can be suspended in isopropanol and rapidly swirled at room temperature. Progressive addition of chloroacetic acid and NaOH solution and reaction carried for 3 h at 60 °C. Finally, the process was culminated by neutralization achieving neutral pH under room temperature. The formed derivative can be concentrated, dialyzed, precipitated, and lyophilized to powder (Wang et al., 2016). The degrees of substitution (DSs) can be calculated by acid-base titration (Liu et al., 2021). Water insolubility of many polymers makes them harder to produce bioactivities, carboxymethylation is a method of transforming their nature and enhancing their solubility, bioactivities, and anti-proliferation of cancer cells (Kagimura et al., 2015). Carboxymethylation significantly influenced water solubility, surface charge, reduction in viscosity (Andrew and Jayaraman, 2020), and lowered gelation temperature of polysaccharides (Tranquilan-Aranilla et al., 2012). Such derivatives possess potential uses in the food, pharmaceutical, chemical, and cosmetic sectors (Kagimura et al., 2015). α-D-glucans after carboxymethylated depicted enhanced biological activities that could be considered for therapeutic dietary supplements (Wiater et al., 2012a).

#### 3.4.4 Phosphorylation

**Fig. 4:**
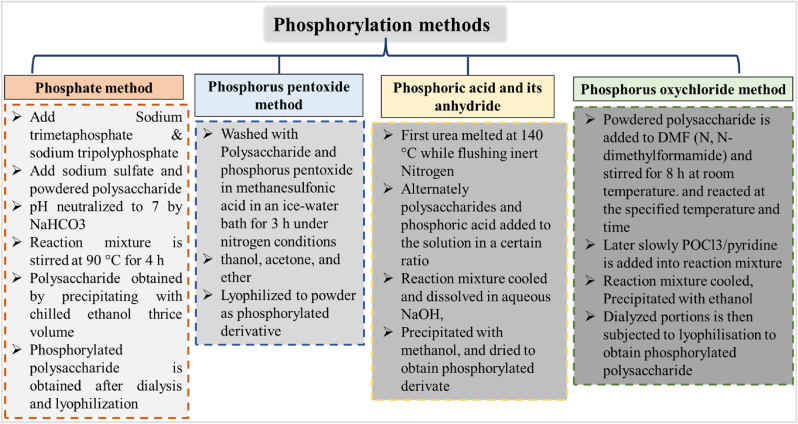
Phosphorylation methods for bacterial EPS and steps.

Phosphorylation is one more approach to replace hydroxyl groups with the incorporated phosphate group in polymer chain configuration (Ahmad, 2021). Phosphorylated polysaccharides are uncommon in nature. Monosaccharide like glucose, fructose that are bereft of bioactivities can be activated by phosphorylation (Xia et al., 2021). Phosphorylation can be achieved through methods involving phosphate, phosphorus pentoxide, phosphoric acid and their anhydride as depicted in (Fig. 4**)** (Chen et al., 2019; Xie et al., 2020; Hu et al., 2020). This procedure possibly enhance the polysaccharide solubility in water, allocate electron donation, possess nucleophile characters, modifying molecular weight and improved chain conformation (Andrew and Jayaraman, 2020). Such modification have caught attention due to their potential antioxidant, antiviral, antitumor, immunomodulatory and hepatoprotective properties (Xiong et al., 2019; Feng et al., 2017). The glucan polysaccharide from *Lachnum* sp. was subjected for phosphorylation the structural integrity remains unchanged. However, results in-vivo mice models suggested that antitumor activity was significantly improved with insignificant side effects. Increase in rate of tumor inhibition showed dose activity relationship varies from 53.02 % to 60.85 % for unmodified to 66.1% - 68.57 % for phosphorylated polysaccharide at dose of 100 mg/kg and 400 mg/kg respectively. Further, phosphorylated lachnum polysaccharide had more inhibitory effects on S180 sarcoma with enhanced TNF-α, SOD and IL-2 suggesting antitumor potential (Ye et al., 2013).

#### 3.4.5 Benzoylation

**Fig. 5:**
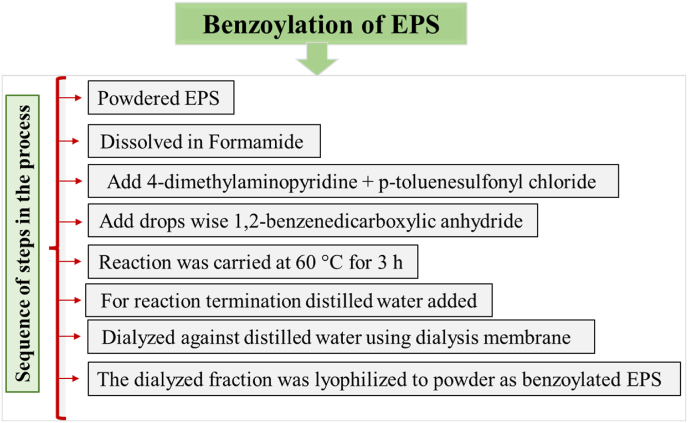
Sequence of steps in the benzoylation process for EPS modification.

EPS secreted by *Paenibacillus polymyxa* EJS-3 was benzoylated according the method reported by (Liu et al., 2012) and the sequence of steps followed during the process of derivitisation is presented in (Fig. 5). Introduction of benzyl groups into EPS composed of levan increased antioxidant and antitumor activities, increment in bioactivity can ascribed to electron donating potential and affinity towards the immune cell receptors (Liu et al., 2012). Compounds' antioxidant properties have been linked to a variety of methods, including the ability to reduce substances, capability to scavenge free radicals and form bonds with transition metal ion catalysts. Such properties could be possible indicators for clueless antioxidant potential after these incorporated groups. Levan benzoylated derivative's stronger reducing power than native form suggested that the antioxidant activity had been significantly increased through chemical alterations. However, were inferior to that of vitamin C (1.2) (Liu et al., 2012). Reducing power was generally attributed to its electron-donating ability. The superoxide is considered as key primary ROS and thought to be produced during irradiation from oxygen or through metabolic activity. Secondary ROS, such as the singlet oxygen, hydrogen peroxide, and hydroxyl radicals can be generated by additional interactions between the superoxide radical and other molecules, either directly or more frequently through enzyme- or metal-catalyzed reactions that have potential to cause the protein, lipid and DNA damage.

Benzoylated levan EPS at 1.0 mg/mL showed superoxide scavenging capacity (89.8 %) more than two times than unmodified levan (40.4 %). However, at the same concentration vitamin C depicted substantially much lower activity 12.11 % (Liu et al., 2012). Practically every bio-macromolecule in live cells is susceptible to attack and destruction by the most reactive radical known as hydroxyl radical. Its ability to promote lipid peroxidation with the attack on cell membranes and side chain of fatty acids of phospholipid membranes. Levan EPS at 1.0 mg/mL showed hydroxyl scavenging capacity (68.47 %) which is lower than its benzoylated derivative (72.37 %) (Liu et al., 2012). The outcomes suggested that levan's EPS capacity to scavenge hydroxyl radicals may be increased by adding chemically modified groups. The in-vitro studies conducted by the researchers have found that levan EPS after benzoylation provide activity against BGC-823 (human gastric cancer cells) and observed that at a dose of 400 μg/mL, inhibition of 91.67 % was recorded which is significantly higher than unmodified levan (55.37 %). However, compared to cisplatin inhibition rates was (96.23 % at 5.0 μg/mL) that is significantly higher than levan and its derivative (Liu et al., 2012). The second greatest cancer-related mortality rate worldwide in recent years is due to stomach cancer. In view of that, more focus has been placed on creating specific compounds that can be used selectively to stimulate terminal differentiation, which would eventually result in the removal of tumor-causing cells and the restoration to typical cell homeostasis. There are scarcity of reports on benzoylation and its impact on activity of bacterial exopolysaccharides. Therefore, it is imperative that scope of benzoylation can be further broadened for the existing and future exopolysaccharide applications.

#### 3.4.6 Selenization

**Fig. 6:**
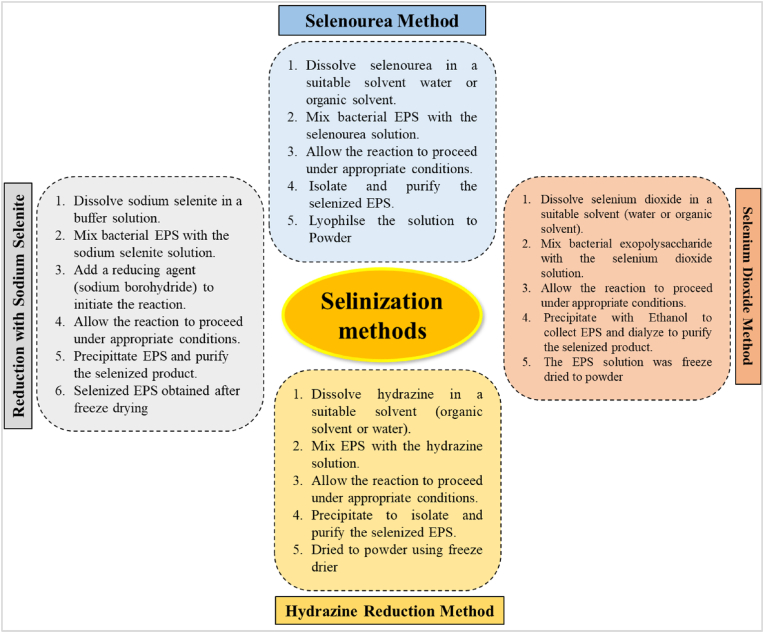
Sequence of steps in selinization process for EPS modification.

Selenium a critical trace element, is crucial for many aspects of both human and animal health. Recent research suggests that selenium can enhance the biological effects of polysaccharides. In humans, glutathione peroxidase and superoxide dismutase are enzyme systems that contain the trace element selenium as a component. The inhibition and eradication of free radicals can be catalyzed by glutathione peroxidase with the help of selenium. Various procedures illustrated to synthesize the selenylated EPS (Cheng et al., 2018) as depicted in (Fig. 6). Selenide polysaccharides are produced when selenite or selenious acid forms selenite ester linkages with the hydroxyl groups on polysaccharides in suitable conditions (Li et al., 2016). Selenium levels in natural polysaccharides are typically low, ranging from very few to tens of μg/g.

However, reports have demonstrated that up to several hundred milligrams of selenium can be introduced during fermentation of microorganism in selenium enriched media typically containing up to 560–3300 μg of selenium per gram (Li et al., 2020). The EPS built of monomers of glucose, galactose, and mannose from *Enterobacter cloacae* Z0206 after selenium modification enhanced the bioactivity with improved innate, cellular, and humoral immunity in mice that have undergone cyclophosphamide-induced immunosuppression. Besides that, immunomodulatory activity has been established in crayfish, mice, and broilers. The activities of SOD and GPx were dramatically increased by the administration of se-EPS at concentration of 560, 840, and 1120 mg/kg, and the serum concentration of malonaldehyde (MDA) a marker of lipid peroxidation was significantly decreased in the study carried for broilers (Lu et al., 2013). In another investigation, EPS produced by *Bacillus licheniformis* CGMCC 2876 in a medium based on waste cane molasses. EPS was successfully used to synthesize a novel form of organic EPS selenium nanoparticles during the green synthesis (Wang et al., 2023). These EPS-selenium nanoparticles demonstrate potential antioxidant activity in scavenging superoxide, hydroxyl, and DPPH free radicals within a concentration of 0.25–4.0 mg/mL, inhibiting nearly up to 90 %. Furthermore, during in-vitro studies on intestinal pathogenic gram-positive and gram-negative bacteria the EPS-selenium nanoparticles could not allow growth more than 80% at a dose of 1000 μg/mL due to their outstanding antibacterial action (Wang et al., 2023). EPS from *Bacillus* sp. MKUST-01 was synthesized to EPS-selenium nanoparticles isolated from the silt soil of the Manakudi Estuary in the Kanyakumari District of Tamilnadu, India (Ramachandran et al., 2023). These selenium nanoparticle-EPS conjugates performed 20% better than selenium nanoparticles and EPS alone in terms of antioxidant activity. In addition, EPS-selenium nanoparticles conjugate correlated with higher growth and increased survival rates in *Artemia nauplii* larvae (model organism for assessment of toxicity) compared to diet fed with selenium nanoparticles and a microalgal diet (Ramachandran et al., 2023). EPS based selenium nanoparticles synthesized from *Lactococcus lactis* subsp. *lactis* demonstrated immunomodulatory effects on spleen lymphocytes and peritoneal macrophages. Secretions of nitric oxide (NO), IL-1, IL-6, IL-12, and IFN-γ were all up, though not IL-10 levels in mice peritoneal macrophages. Selenium-EPS demonstrated stronger immunomodulatory activity than EPS which is revealed during the initial experiments of enhanced proliferation of mouse spleen lymphocytes and markedly increased levels of IL-2, IL-6, and TNF- in spleen cells These results suggest that EPS and Selenium-EPS can enhance the immunological response via spleen lymphocyte stimulation and enhanced macrophage (Pan et al., 2015). More research is required to explore the reported dose impact and identify the immune-functional effects and mechanisms of selenium-EPS modifications.

## 4 Role of modified EPS in human health

The addition of a functional chemical group to the polysaccharide changes the structural nature and eventually alters the functional properties. Following are a few biological properties influenced by the addition of various chemical groups with chemical modification methods.

### 4.1 Role as antioxidants

In the food sector, antioxidants are important for preservation, flavor, shelf life, and nutrition consistency. Natural antioxidants, on the other hand, are considered to be beneficial in curing diseases and reducing free radical damage. As a result, natural antioxidants will become increasingly important in the future for preservatives, medical research, additives, and functional foods. Free radicals cause oxidative damage, which is an inevitable and effective process that causes cell damage and results in diseases including cancer, diabetes, and cardiovascular diseases (Rudrapal et al., 2022). Under such circumstances, antioxidants have an essential role to play in postponing or reducing adversity of oxidative havoc caused to cells by free radicals. EPS from various microbial sources post-modification displayed strong protection, even at low concentrations, due to their unique structural features as presented in Table 2**.** The prevalent use of synthetic antioxidants like BHA (Butylated hydroxyanisole) and BHT (Butylated hydroxytoluene) are suspected for carcinogenesis and liver ailments. In view of that, it happens to be a daunting task to search for and develop some natural radical scavengers to protect human health and prevent from chronic progression of diseases. Phosphorylated EPS generated by *Lactobacillus lactis* subspp *lactis* showed significant antioxidant and scavenging action, notably on superoxide, hydroxyl, and DPPH radicals (Guo et al., 2013). Acetylated polysaccharides can contribute more electrons to the radical chain reaction slowing it down (Wang et al., 2022). EPS generated by *Streptococcus thermophilus* using glucose and galactose as sugar units have demonstrated greater scavenging capabilities after sulfation (Li and Shah 2016). EPS produced by *S.thermophilus* GST-6 upon sulphonation exhibited stronger antioxidant properties than the unmodified EPS (Zhang et al., 2016). Sulfated EPS from *Enterobacter cloacae* showed a noticeable scavenging effect against the superoxide radical and hydroxyl radical (Jin et al., 2011). Selenium-polysaccharide with a larger uronic acid concentration and sulphate have better DPPH, superoxide, and radical scavenging capacity than regular EPS (Zhang et al., 2020). Se- EPS scavenged hydroxyl (-OH) superoxide anion radicals in mice livers, as well as increasing catalase, super dismutase, and glutathione peroxidase activity and decreasing monoaldehyde levels in the blood (Guo et al., 2013). Superoxide anion formed from the electron transport chain of mitochondrial system has been considered as initiating free radical for synthesis of hydroxyl radical, hydrogen peroxide and singlet oxygen for damage of cells in living systems. These sulphate groups improve bioactivities and can suppress the T lymphocyte virus, making sulfated modification an essential avenue for structural alterations of polysaccharides (Liu et al., 2023).

### 4.2 Role as anti-tumorigenic agent

Globally, the incidence of colon cancer ranked third which arises from the inner wall of large intestine pervade grim for human life. Although, chemotherapy is considered the most promising curative strategy for cancers in spite of being extremely toxic and low return therapeutic. Many EPS types derived from natural sources have been explored for their ability to prevent colon cancer. The findings indicated that these EPSs, when taken into account with their inhibitory effects on cell viability (17.2–19.2%) on HCT116, had substantial antitumor benefits (Zhang et al., 2019). However, more specifically the role of synthesized EPS derivatives in terms of antitumor potential has been deliberated. Wang et al. (2015) reported the EPS from *Lactobacillus plantarum* 70810 after being modified to acetylated, carboxymethylated, and phosphorylated forms proved to be more efficient and inhibitory to HepG-2 (human liver cancer) and HT-29 (colon cancer). Percent inhibition was found to be dose-dependent between (50–600 μg/mL), at a maximum concentration of 600 μg/mL inhibition percentage against HepG-2 cells varies for instance unmodified EPS (22.84 %), acetylated EPS (31.75 %), phosphorylated EPS (43.75 %) and carboxymethylated EPS (44.19 %). They also observed that phosphorylated and carboxymethylated derivatives showed significant inhibitory effects (46.11 %) at 600 μg/mL against HT-29 cells, which was almost comparable with 5-flourouracil at a concentration of 50 μg/mL (Wang et al., 2015). According to studies by (Liu et al., 2012), phosphorylated polysaccharides had greater antitumor activity that can be due to phosphate groups affinity for immune cell receptors and help in immune system activation. Besides, carboxymethylated polysaccharide-protein complexes demonstrated more strong in vitro anticancer activity than the unmodified form. EPS from *Lactobacillus helveticus* MB2 exhibited anti-proliferative efficacy against colon cancer cells (HT-29). Anticolon cancer cell activity can be ascribed to augmented LDH leakage, control of G1 to S transition and the initiation of apoptosis through mitochondria and ROS dependent mechanism (Xiao et al., 2020). A novel pentasaccharide from *Lactobacillus acidophilus* 20079 presented promising tumor suppression effect against CaCo-2 colon cancer cells (El-Deeb et al., 2018). Naturally existing carboxymethylated and sulfated EPS secreted from *Halogeometricum borinquense* 52 demonstrated antifatigue activity by gauging mice endurance for the forced swimming. Besides that, antitumor effects were observed against myelogenous leukemia cells and liver human cancer cells (Chouchane et al., 2020). These studies suggest EPS can be passed down as effective food for therapeutics of cancer, particularly human colon cancer.

### 4.3 Role as anti-inflammatory agents

Nonsteroidal anti-inflammatory agents like acetyl-salicylic acid cause stomach cancers and gastritis upon wide use. Exopolysaccharides produced by *Streptococcus thermophilus* strains prevented the chronic gastritis in in-vivo models. EPS secreted from *Streptococcus thermophilus* CRL 1190 mixed with milk was greatly effective for prevention of gastritis in mice, the result can be ascribed to EPS-protein interaction for gastro protective potential (Rodríguez et al., 2009). In most populations, coeliac disease (an autoimmune enteropathy) affects 1 % of populations, is triggered due to gluten consumption, leading to impaired absorption and inflammation in small intestines (Lebwohl et al., 2018). However, lactobacilli EPS can be replaced as gluten-free substance in the development food products with improved techno-functional and health benefits (Ruhmkorf et al., 2012).

### 4.4 Role in cardiovascular health (CVD)

Metabolic syndrome is described by obesity, dyslipidemia, insulin resistance, and hypertension that can increase the risk of cardiovascular diseases (CVD) (Silveira Rossi et al., 2022). Worldwide CVDs are the leading cause of death (Chopra et al., 2022). Dyslipidemia is considered as potential risk factor for CVD, upsurge of interest in alleviating such diseases is recent call for the researchers. *Lactobacillus paracasei* M7 EPS showed remarkable hypocholestrolemic activity (Bhat and Bajaj 2019). EPS from *Alteromonas infernus* possess heparin-like activity, such low molecular weight molecules were named as heparinoids exhibited with strong anticoagulant activity (Jouault et al., 2001). Various hypoglycemic drugs like sulfonylureas, biguanides, and glucosidase inhibitors witnessed in markets. However, complications and mismanagement related to their use are discouraging, suggesting that additional and novel alternatives might be used. Recently researchers (Xu et al., 2017) reported that EPS secreted by *Lachnum* YM130 and its acetylated and benzoylated derivative have profound effects on diabetic mice model. They observed that significant increase of CAT, GPx, SOD activity and a decline in the level of MDA, AGEs, hs-CRP, sICAM-1, NO, and ET-1 in serum. Furthermore, alleviation in myocardial disorders and cardiac muscle fibers necrosis were observed during histopathological examination in diabetic mice at a dose of 200 mg/kg. Therefore, EPS can prove to be a prospective low-fat source in the incorporated food products to mitigate health issues associated with excess fat intake.

### 4.5 Role as anticoagulant

Heparinoids, or naturally occurring or manufactured sulfated polysaccharides possessing heparin-like biological properties, have been the subject of studies (Jouault et al., 2001). The anticoagulation potential of EPS was evaluated by the testing of sodium heparin using U.S. Pharmacopeia Convention. Plasma clots were exposed to aqueous solutions of EPS at specific concentrations. The percentage of plasma clot lysis were used to estimate the fibrinolytic activity. The studies conducted for both native and sulfated EPS from *Enterobacter* sp ACD2 demonstrated notable anticoagulative effects because coagulation time was postponed beyond 24 h, which surpasses coagulation duration of regular heparin (1.5 h). Additionally, it showed that both plasma clots were 100% lysed, surpassing the fibrinolytic capacity of typical hemoclar (75% lysis of the plasma clot) (Almutairi and Helal 2021). Investigations into sulfated polysaccharides that are either naturally occurring or artificially produced and have biological effects akin to heparin referred to as heparinoids have revealed that the anticoagulant activity of these compounds is partially due to the potent interaction between the negatively charged sulphate groups along with some positively charged peptidic sequences (Yang et al., 2013). Dextran sulphate contains anticoagulant and antilipidemic qualities that can be used to treat lipemia and arteriosclerosis (Guezennec et al., 1998). Jouault et al. (2001) reported that Sulphate esters generated by *Alteromonas infernus* EPS are highly involved in the biological activity as evidenced by the rise in *in vitro* anticoagulant activities. The presence of sulphate esters boosted anticoagulant action, demonstrating that they are crucial to biological activity. However, it was also reported that the existence of anticoagulant characteristics is not required for most of these sulphate families. Numerous sulfated polysaccharides that are anticoagulants (such as fucan, pentosan sulphate, dermatan sulphate, etc.) lack sulphate groups in the 6-position. According to a recent study, despite having a high sulphate concentration, dermatan sulphate that is sulfated in 6-position exhibited completely no anticoagulant action compared to dermatan sulphate with 4-O-sulfated galactosamine possess specificity for HCII (Jouault et al., 2001).

## 5 Structure and function relationship of EPS

Understanding the structure and function relationship of EPS permits researchers to produce biopolymers that perform best in different applications, including food, pharmaceutical, and biotechnology. The diverse structures of EPS influence their functions and rheological properties in dairy products. For instance, high-molecular-weight EPS from *Streptococcus thermophilus* enhances the viscosity of acidified milk, while stiff, linear EPS from *Streptococcus thermophilus* provides greater viscosity than branched, flexible EPS (Zhai et al., 2021). During carboxymethylation, hydroxyl groups of polysaccharides are replaced with carboxymethyl groups. This substitution results in changes to the physicochemical properties, conformations, and primary structure of polysaccharides (Xie et al., 2021). Carboxymethyl dextran benzylamide was reported to inhibit the in vitro cell proliferation of 1205 L-U and 1205 L-U tumor development in athymic mice (Li et al., 2021). The impact of monosaccharides on polysaccharide bioactivity has been extensively studied due to their effectiveness against bacteria, inflammation, and tumors, with little side effects. Reports showed that glucose mainly affects their prebiotic activity, arabinose and xylose have an influence on immunomodulatory and prebiotic potential. Fucose and sulphate have a role in anti-inflammatory activity; rhamnose affects antibacterial and antioxidant properties, and uronic acid has significant antitumor activity likewise mannose has immunomodulation potential (Wang et al., 2024). Infernan, an EPS from bacterial strain GY785, has a complex repeating unit of nine monosaccharides on a double-layer of side chains. Sulfated and uronic chains are attributed to its functional and biological properties. The presence of calcium binding sites (Ca ^2+^) sites further infers the formation of gels which are essential for various technological and biological aspects (Makshakova et al., 2022). EPSs can form hydrogels and retain large amounts of water inside their cross-linked 3D network structures while remaining insoluble, allowing them to entrap biomolecules (drugs) and adsorb them onto their external surfaces, prolonging drug residence, increasing drug bioavailability, and lowering drug cytotoxicity (Daba et al., 2021). The molecular arrangement and primary structure of EPS secreted from *Colwellia psychrerythraea* 34H, which is a cold-adapted bacterium, have a significant role in ice recrystallization inhibition. The prevention of ice recrystallization may be due to a pseudohelicoidal structure that hinders the native tetrahedral order of the water molecules in the initial hydration shell and has potential scope in cryoprotection in foods, medicines and biotechnological applications (Casillo et al., 2017).

## 6 Safety of chemical derivatives and way forward

Safety assessment of chemically modified EPS is crucial to ensure their suitability for various applications, though EPS is regarded as safe, biocompatible, biodegradable, and non-toxic to humans or animals (Moscovici 2015). Modified EPS derivatives like carboxymethylated, acetylated, and phosphorylated that are originally obtained from *Lactobacillus plantarum* showed no effect on the proliferation of human embryo kidney 293 cells i.e.; human normal cells (Wang et al., 2015). EPSs synthesized from natural sources deliver many advantages over synthetic drugs which despite being efficient frequently display lethal activity toward host cells. The possibility of lessening the deleterious effects of synthetic chemotherapeutics can be achieved by agglomeration of natural substances that may enhance the potential and could be quite promising, but warrants careful consideration. However, there are some key considerations that need to be addressed: Firstly, chemical residues and impurities during the synthesis process like solvents, impurities, and residual reagents should be ensured within acceptable limits. Second, toxicity studies should be conducted through appropriate models and assays. Third, ensure compliance with regulatory standards for safety and use in relevant industries (e.g., FDA, EU, FSSAI regulations). Fourth, benefit analysis to weigh the potential benefits of using the modified EPS against any identified risks to determine if the benefits outweigh the potential hazards. Fifth, need to be assessed the potential for allergic reactions in humans or animals for in vivo studies. Therefore, it is important to note that specific safety evaluation protocols may vary depending on the intended application of the modified EPS (e.g., food additives, pharmaceutical excipients, biomedical materials).

## 7 Conclusion and future perspective

This review offered proof that enhancing the bioactivity of EPS through chemical modification might be a successful strategy. Increased oxidative stress combined with an unhealthy lifestyle is considered a key contributor to the emergence of diseases and a burden on human health. Therefore, there is a need to supplement the protective molecules of natural origin with less or no involvement of chemical entities to address oxidative stress. Antioxidants have the potential to combat the free radical production machinery. However, having natural antioxidants in place is very crucial and that too holds renewable, biocompatible, and sustainable status. It is well documented that the structural properties of EPS possess various roles in food and biomedical applications and that EPSs contribute a greater share of natural hydrogen to deliver bioactivity. Their perceived biodegradable, non-toxic, biocompatible, and thermally stable nature could further add value to their applications. It is inevitably achievable to raise the physiochemical features of EPS by playing with its chemistry, particularly through various modifications as discussed in this review. Based on their molecular makeup or composition, all the EPS addressed in this research have demonstrated substantial antioxidant properties. The review included information supporting the possibility that the bioactivity of EPS could be enhanced through chemical modification. Additionally, the findings may help determine the course of molecular modification and serve as a basis for the creation of de novo food additives. Furthermore, these compounds may be examined as novel prospective antioxidants and cancer-fighting compounds. The reviewed literature suggested the existence of a stronger relation between structure and functional activity of polysaccharides. However, additional research is required to explore their safety and activity in vivo models, which can expand their wide relevance at a larger scale.

## Authors contribution

Irshad Ahmad Shah: Conceptualization, original draft writing, and editing. Digambar Kavitake: Conceptualization, writing, review and editing. Swati Tiwari: Writing, review and editing. Palanisamy Bruntha Devi: Writing, review and editing. G. Bhanuprakash Reddy: Review, editing and supervision. Krishna Kumar Jaiswal: Writing, review and editing. Amit K. Jaiswal: Conceptualization, review, editing and supervision. Prathapkumar Halady Shetty: Conceptualization, review, editing and supervision.

## Declaration of competing interest

The authors declare that they have no known competing financial interests or personal relationships that could have appeared to influence the work reported in this paper.

## Data Availability

No data was used for the research described in the article.
